# Barriers and facilitators in developing patient versions of clinical practice guidelines - qualitative interviews on experiences of international guideline producers

**DOI:** 10.1186/s12913-023-10524-5

**Published:** 2024-01-16

**Authors:** Nora Meyer, Julia Hauprich, Jessica Breuing, Irma Hellbrecht, Sarah Wahlen, Nadja Könsgen, Stefanie Bühn, Monika Becker, Susanne Blödt, Günther Carl, Markus Follmann, Stefanie Frenz, Thomas Langer, Monika Nothacker, Corinna Schaefer, Dawid Pieper

**Affiliations:** 1 Institute for Research in Operative Medicine (IFOM), Department for Evidence Based Health Services Research, Department of Medicine, Faculty of Health, Witten/Herdecke University, Ostmerheimer Str. 200, Building 38, 51109 Cologne, Germany; 2 Institute for Medical Knowledge Management c/o Philipps University Marburg, Association of the Scientific Medical Societies in Germany, Marburg/Berlin, Germany; 3 German Prostate Cancer Support Group, Bonn, Germany; 4 Office of the German Guideline Program in Oncology (GGPO),c/o, German Cancer Society, Berlin, Germany; 5 Frauenselbsthilfe Krebs Bundesverband, Bonn, Germany; 6 German Agency for Quality in Medicine, Berlin, Germany; 7 Institute for Health Services and Health System Research (IVGF), Brandenburg Medical School Theodor Fontane, Rüdersdorf bei Berlin, Germany

**Keywords:** Patient versions of clinical practice guidelines, Methodology, Development, Dissemination, Implementation, Evidence-based patient information, Guideline development

## Abstract

**Background:**

Several guideline organizations produce patient versions of clinical practice guidelines (PVGs) which translate recommendations into simple language. A former study of our working group revealed that few guideline organizations publish their methods used to develop PVGs. Clear definitions of PVGs do not prevail and their purposes often remain unclear. We aimed to explore experts’ perspectives on developing, disseminating and implementing PVGs to discuss and incorporate these experiences when consenting on methodological guidance and further improving PVGs.

**Methods:**

We conducted 17 semi-structured telephone interviews with international experts working with PVGs from September 2021 through January 2022. We conducted the interviews in English or German, they were recorded and transcribed verbatim. We utilized Mayring’s qualitative content analysis with MAXQDA software to analyze the data.

**Results:**

In two interviews two participants were interviewed at the same time. This resulted in a total of 19 participants from 16 different organizations and eight different countries participated. Most were female (16/19) and their experience in working with PVGs ranged from 1 to 20 years. All follow methodological standards when developing PVGs, but the extent of these standards and their public accessibility differs. Aims and target groups of PVGs vary between organizations. Facilitators for developing PVGs are working with a multidisciplinary team, financial resources, consultation processes and a high-quality underlying CPG. Facilitators for disseminating and implementing PVGs are using various strategies. Barriers, on the other hand, are the lack of these factors. All participants mentioned patient involvement as a key aspect in PVG development.

**Conclusion:**

The steps in the PVG development process are largely similar across the countries. Focus is placed on the involvement of patients in the development process, although the extent of participation varies. The experts collectively attribute great importance to PVGs overall, but in order to constantly adapt to medical progress and changing conditions, the focus in the future may be more on formats like living guidelines. Although there are different views on the mandatory development of PVGs, there is a consistent call for more transparency regarding the methodology used for PVGs.

**Supplementary Information:**

The online version contains supplementary material available at 10.1186/s12913-023-10524-5.

## Introduction

Clinical practice guidelines (CPGs) contain medical recommendations primarily designed to assist clinicians and other professionals in providing care [1, 2]. Several guideline organizations produce patient versions of clinical practice guidelines (PVGs) which translate these recommendations into simple language [3]. Such evidence-based patient information (EBPI) is intended to enhance health literacy as well as informed decision-making and can strengthen patient-physician communication, as CPGs and parallel PVGs share the same information base [4]. Reporting on the methodology of PVG development may be useful for patients in terms of credibility of information and for developers in terms of continuous improvement of PVGs.

Although there are already a variety of organizations producing PVGs, clear definitions of PVGs do not prevail, their purposes often remain unclear, and there is a lack of differentiation from other patient information tools [5, 6]. In a recent scoping review, we found that some guidance documents on developing PVGs already exist [4, 5, 7] but are not yet widely implemented and followed and there is not yet an international consensus on basic methodological aspects in the development of PVGs [8]. Not many guideline organizations publish their methods used to develop PVGs as method reports, however, we assume that there are indeed internal standards within organizations [8]. Thus, the consultation of experts involved in the development of PVGs is a further logical step to obtain information which can be incorporated in the further improvement of PVGs.

By conducting semi-structured interviews with national and international experts in the field of PVGs, we aim to:

1. Obtain information on the methodological approach in the development of PVGs.
2. Gain insights into the experiences of working with PVGs, in particular with regard to factors that promote and hinder the development, dissemination and implementation of PVGs.

## Methods

This study is part of a large multi-phase study (AnImPaLLO project) investigating the (inter-)national role and applicability of PVGs in order to derive recommendations for the development, dissemination, and implementation of PVGs in Germany. The detailed methods are reported elsewhere [9]. We followed the consolidated criteria for reporting qualitative research (COREQ) checklist to report our study (Appendix A) [10].

### Study population and recruitment

We planned to conduct interviews with the international organizations from a study on the content and purpose of PVGs by Santesso et al. [11] and with national experts. We recruited until we reached data saturation. The study was approved by the Witten/Herdecke University Ethical Committee (160/2021). We obtained written informed consent from all participants. There was no incentive for participation.

As several organizations in Germany produce PVGs, we aimed to conduct at least three interviews with experts in this field. We contacted five medical societies from the database on PVGs by the Association of the Scientific Medical Societies in Germany (German abbreviation: AWMF) that published the highest number of PVGs since January 1st, 2018 and additional organizations recommended by the German Guideline Program in Oncology (GGPO), as the response rate was rather poor.

On the international level, the 17 guideline organizations referred to in Santesso et al. [11] were contacted and invited to participate in the telephone interviews. Since the response to our invitation was low, we decided to contact additional guideline organizations. To do so, we contacted the organizations producing EBPI mentioned in a paper by Van der Weijden et al. Further, we searched the GIN Members Directory. Organizations that had previously been contacted as part of the organizations from Santesso et al. and the German organizations involved in the research project (AWMF, ÄZQ and GGPO) were excluded. As of October 2021, a total of 110 GIN members remained and from this list, ten guideline organizations were randomly selected and invited to participate in the interviews. Further organizations or individuals were contacted upon recommendations from other experts or the cooperation partners within the project. We contacted organizations that we knew to produce PVGs and that we would consider as such, as they were produced on the basis of a CPG and thus met the most important criterion in our view. However, we did not ask for a clear definition of PVGs in advance.

We invited all guideline organizations via email and sent reminders approximately four weeks after the initial contact. We requested to name a representative of the respective organization willing to participate in an interview. Next to expertise in the development of PVGs, the interview participants needed to be ≥ 18 years old and proficient in either German or English language.

**Fig. 1:**
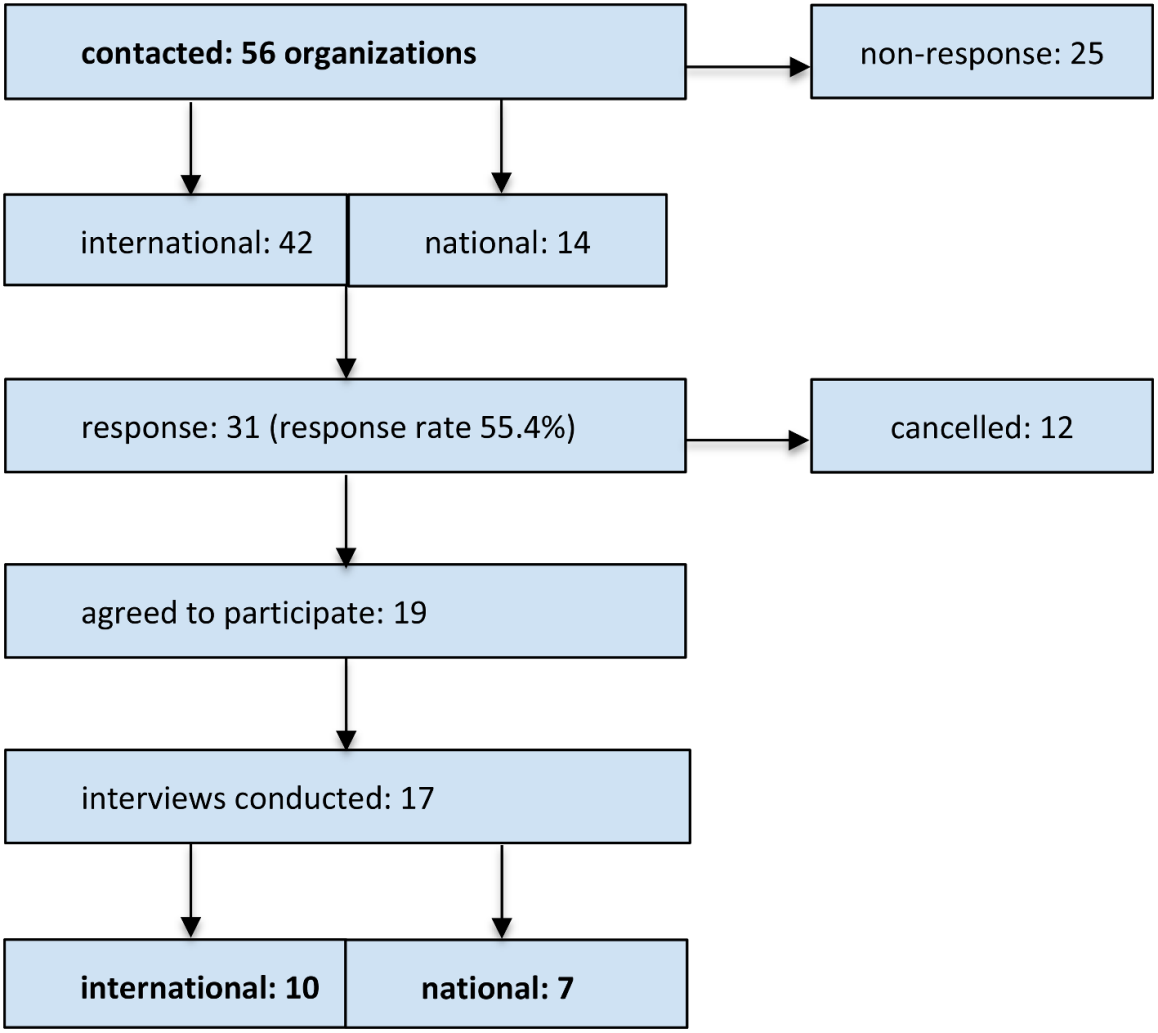
Flow chart of the recruitment process

In total, 56 national and international organizations or individuals were contacted, 25 did not respond at all, even after sending reminders. Twelve organizations canceled because they could not find the personal capacity for an interview, or because they weren’t involved in PVGs. A total of 19 agreed to participate in an interview, however two did not get back to us after consenting, so in the end, we conducted a total of 17 interviews. Figure 1 shows the recruitment process.

### Interview guide

The systematic literature search on the applicability of PVGs conducted as part of an earlier module of the overall project [9] provided insights that contributed to the development of the interview guide. In addition, the project partners involved in the development of the interview guide include people with extensive expertise both in the development of PVGs and in conducting qualitative interviews. We designed the interview guide in German and sent the draft to the project partners, who provided thematic support. After including amendments, we translated the final interview guide into English language. Both interview guides were pretested using the first English and the first German interview as pretest and complementing the interviews accordingly, if necessary. The final interview guide in English is provided in appendix B. It queries personal information about the interviewee like gender, age, occupation and years of experience in the development of PVGs. We then asked about aims, formats, target groups and usual topics of PVGs. The focus was on the development process of PVGs and challenges occurring alongside as well as on the public availability of methods on PVGs and whether these are presented in a way understandable to laypersons. Lastly, we queried facilitators and barriers in the development, dissemination and implementation of PVGs and requested perspective on the future importance of PVGs, especially in the context of demographic change and digitalization.

### Data collection

The interviewer (NM), who is a female health scientist, had little experience in conducting qualitative interviews but was trained in advance. The participants did not know the interviewer (NM), they only knew she was a researcher at Witten/Herdecke University. NM conducted the interviews either by telephone or ZOOM [12] video conferencing, depending on time zones and the individual preferences of the interviewees. The interviews were conducted either in English- or German-language. The interviews were recorded using an audio recording device and were transcribed verbatim by an external institution [13]. In one case, transcripts were returned to participants because participants in a dual interview wanted to check for any misunderstandings that might have occurred due to the language barrier. Otherwise, no transcripts were sent for comment. No field notes were made during the interview. No interviews were repeated and none had to be canceled. The participants completed an informed consent form and a data protection statement and were provided with the interview guide in advance. Names of the interviewees and their corresponding institutions are kept confidential. For quotes, the corresponding passages from German-language interviews are translated into English using DeepL [14].

### Data analysis

Sociodemographic data of the interviewees were descriptively processed using Excel 2016 and qualitative content analysis [15] were performed using MAXQDA Software [16]. NM structured interview data deductively according to predefined main categories based on the core questions of the interview guide. JH then inductively refined the scheme with additional categories and subcategories. NM and JH continuously discussed the results with each other. Disagreements were resolved with a third person (JB). Frequencies were presented as a word cloud using MAXQDA to create a visualization of quantifiable data where appropriate. Based on the interview guides, data codes were defined as follows:

1. General information on the interviewee.
2. General information on PVGs.
3. Target Group.
4. Methodology of PVGs.
5. Future of PVGs.
6. Influential factors on PVGs.
7. Changes on PVGs.

In addition, rules for coding and code specifications were defined (Appendix C).

## Results

### General information

#### General information on the participants

We conducted a total of 17 telephone or internet-based interviews from September 2021 through January 2022. In these 17 interviews, 19 participants from 16 different organizations and eight different countries participated. We conducted two interviews independently with members of one institution, whereas two other interviews were conducted with two participants each instead of one. This happened upon request of the interviewees, either to bridge a language barrier or supplement each other’s answers. In one of these interviews where a possible language barrier was present, the interview transcript was returned to the participants for correction, but no amendments were made. Table 1 shows the demographic characteristics of the study participants and their experience in working with PVGs. Of the 19 individuals, two were male, 16 female and one did not answer the question. The participants were between 26 and 63 years old. All of them had an ISCED Score > 5 on the International Standard Classification of Education [17]. Their experience in working with PVGs varied between one year to twenty years of experience. While some were involved strongly in the development of PVGs, others were more involved in proofreading the final PVGs. This is why the number of involved PVGs ranged from one to up to 700. In 17 interviews, seven different terminologies for “patient version of clinical practice guidelines” were identified. The average duration of the interviews was 48:43 min (range 27:41 min to 01:19:56).

**Table 1:** Participants’ characteristics

ID	Country	Gender	Age [years]	Patient versions*	Experience with patient versions°	Terminology
ID_1	UK	female	50–59	50–100	20	Information for the public
ID_2	Germany	female	60–69	n.s.	n.s.	Patientenleitlinie (“patient guideline”)
ID_3	USA	male	50–59	10	11	patient and family summaries
ID_4	Australia	female	50–59	40	7–8	consumer information / parent information
ID_5.1 ID_5.2	Spain	female female	40–49 40–49	10 1	n.s.	Patient version
ID_6	Germany	female	40–49	1	n.a.	Patientenleitlinie (“patient guideline”)
ID_7	Germany	female	50–59	2	10	Patientenleitlinie (“patient guideline”)
ID_8	USA	n.s.	n.s.	40–50	15	patient summaries / patient information
ID_9.1 ID_9.2	Canada	female female	20–29 20–29	3 2	3 2	patient knowledge translation tools
ID_10	Germany	female	30–39	2	1	Patientenleitlinie (“patient guideline”)
ID_11	Germany	female	40–49	2	1	Patientenleitlinie (“patient guideline”)
ID_12	UK	female	40–49	30–40	14	patient versions of guidelines / public version
ID_13	Belgium	female	50–59	700	2	Guides-patients (“patient guide”)
ID_14	Germany	female	40–49	10–11	10	Patientenleitlinie (“patient guideline”)
ID_15	USA	female	40–49	4	9	patient version / patient page
ID_16	Germany	female	40–49	3	3	Patientenleitlinie (“patient guideline”)
ID_17	Colombia	male	40–49	50	5	Guía para el paciente (“patient guide”)

* Number of patient versions involved in (any kind of involvement), ° years of experience in working with patient versions

#### Aims, contents and formats of PVGs

The experts listed a variety of aims of PVGs, primarily to inform and educate patients on the specific disease. They mentioned the improvement of health literacy and decision-making as a main aim of PVGs. Further aims were to raise awareness of the underlying CPG, give patients confidence and improve self-management of the disease, but also support the contents of the CPG, to disseminate and implement evidence-based information and create transparency for medical action.

Thematic contents covered a broad range of different medicine disciplines, such as oncology or neonatal care. Structural contents, like what kind of information is provided, included background knowledge, information on the disease, e.g., epidemiology, diagnostics and therapy to be part of PVGs, just as information on health care structures and patient rights. PVGs may also include tips for self-management and for improving physician-patient communication. Three German speaking experts said that the PVGs follow a uniform structure and base on the respective CPG.

All guideline organizations provided their PVGs mostly as PDF documents. Some international producers also had interactive PVGs such as HTML format, a quiz or apps, whereas the German organizations rather additionally provided them as print versions.

#### Target group of PVGs

Many organizations do not clearly define the target group of their PVGs. Some state that they address PVGs to the general population, to self-help organizations, caregivers or clinicians. In addition, PVGs target individuals affected by certain diseases and their relatives. Some respondents indicated that their PVGs are more appropriate for educated readers, while others target their PVGs to readers with varying levels of language proficiency or to readers with low health literacy.

#### Governance of PVGs

Eight of the participants reported that creating a PVG for each clinical guideline was a mandatory component of their program. Mandating the development of PVGs seems to be controversial, as PVGs require a reliable data basis, which may not always be available, and considerable time and financial resources. Mandatory development might also create pressure, which could possibly affect the quality of PVGs. Others mentioned the complex structure of the health care system and the difficulty of implementing an obligation across the board and across organizations.

Yeah, I think it would be useful if it were mandatory, because it means that, you know, we are ensuring that people do get access to the recommendations, and it’s, you know, ensuring fairness and equality. So, I think it would be an advantage to do that. (ID_12, Pos. 93).

I mean, I don’t think we can make anything mandatory, because it all comes down to if there is a need for a specific topic. So, I don’t think we need to go in the direction of mandating some of these things and leave it to say the judgment of the guideline developer whether you do need public guidelines or not. (ID_8, Pos. 77).

### Research question 1: methodology of PVGs

#### Initiation of a PVG

On the one hand, sponsors may demand the development of a PVG on a specific topic or the development of PVGs may be initiated when a law changes and existing information needs to be changed accordingly. The impulse can also be bottom-up, when PVGs on specific topics are requested by patient organizations or clinical networks. Whether a PVG will be developed may depend on the available resources, financial and personal as well as on the social relevance of a topic.

#### Methodological approach

**Fig. 2:**
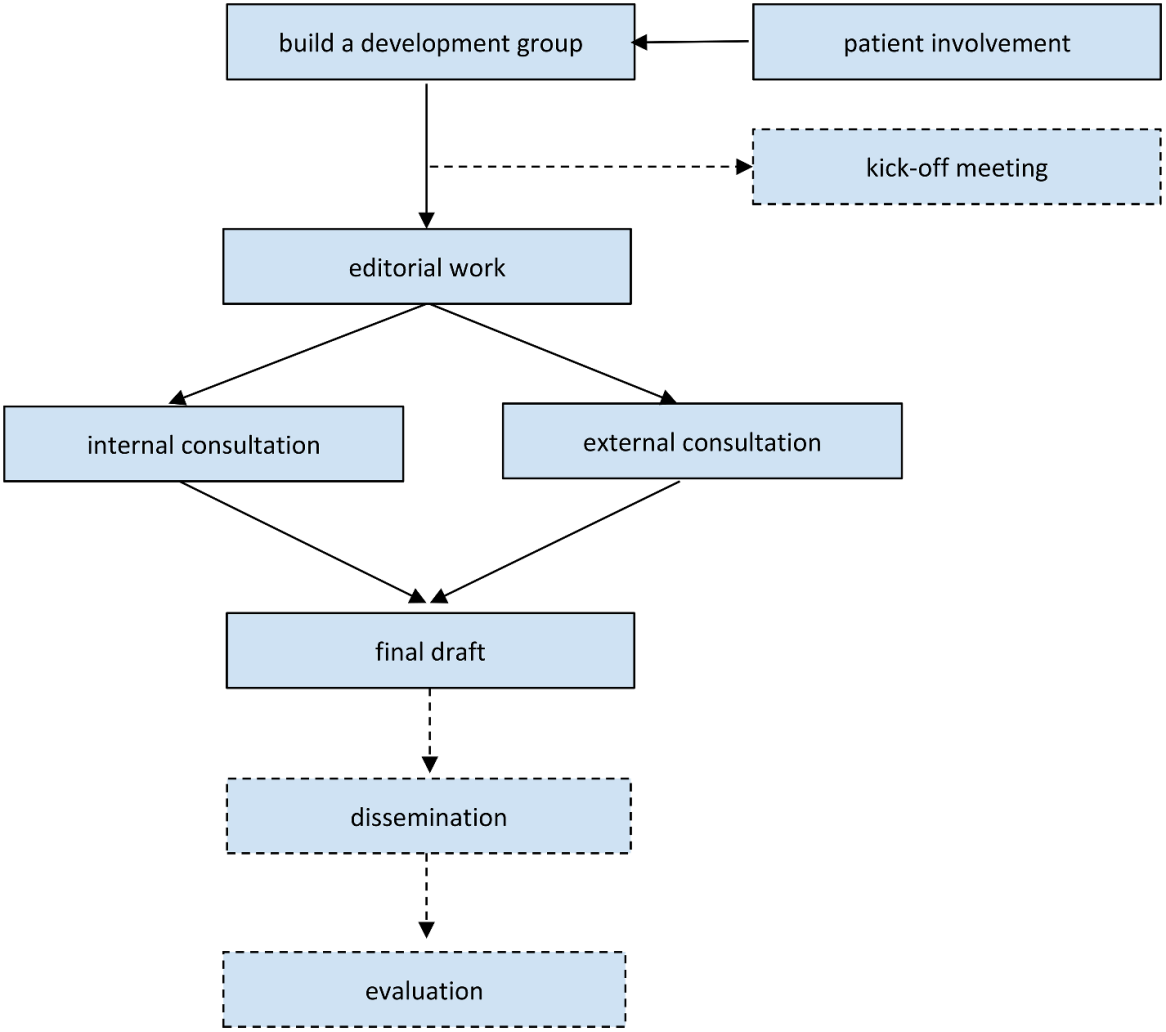
Common steps in developing PVGs

The methodological approach for developing PVGs may vary between organizations, however some steps are quite similar. Figure 2 shows common steps in developing PVGs in a simplified way, where solid boxes show steps that were mentioned by many respondents and dashed boxes show steps that were mentioned less frequently.

The development group consists of stakeholders and usually also includes persons who were already involved in developing the respective CPG. Lay members such as patients themselves, relatives, the public, or patient advocates are usually included in the development process.

(So, what we do is when we’re developing them [PVGs], we take a subgroup from the main guideline group. So, we have the patient carer members of that group, and they’re then part of the development group responsible for the patient versions. So, they’re there every step of the way, they’re involved in choosing the recommendations for inclusion in the patient version, they’re involved in writing over and, you know, advising on language and that sort of thing. (ID_12, Pos. 51).

The German interviewees mentioned a kick-off meeting, where the development group receives a training session. Afterwards, the editorial process starts with writing the first draft. All mentioned that their PVGs are based on the respective CPG, so the development group needs to decide which recommendations from the CPG to include in the PVG or which to prioritize when there is a high number of recommendations.

After incorporating the feedback from the consultation phase, the final version can be sent to the members of the development group, participating organizations and sponsors for acknowledgement and the PVG can be published and disseminated. Likewise, through using a variety of dissemination channels such as journal submission, social media, websites, conference presentations, or dissemination via email distribution lists and notification of key stakeholders. The PVG may be evaluated by conducting surveys, usability testing or online analytics and - if resources allow - by conducting an implementation study as a funded project.

All but one (ID_5) mentioned the use of consultation processes through internal reviews within the development group and/or external through a public consultation phase, surveys or focus groups. The interviewees underlined the importance of patient involvement, as lay members can be experts for their lifeworld.

(…) even when you have people (…) who are health educators and have expertise in developing materials for the general public having someone who is a consumer to say, how they would interpret what you’re trying to say is very important. (ID_15, Pos. 99).

#### Development standards

All follow some kind of standards when developing PVGs, however, the extent of these standards differs. Table 2 lists the public accessibility and obligation to develop PVGs for all guideline organizations participating in the interviews. Some use templates to structure their PVGs consistently, have checklists for creating PVGs, follow internal directives such as standard operating procedures or specific directives for patient involvement. Others stated that they use external position papers and guidelines such as “Good Practice Health Information“ [18] or information from the “Patient Information Forum” [19] to create PVGs. Most display their methods on their websites, others publish them as reports. Even if it is unknown how and if patients use such publicly available methods, respondents call for transparency with regard to the publication of their methods used.

**Table 2:** Public accessibility and obligation to develop PVGs

ID	methodological standards	public accessibility of methodological standards	is developing PVGs mandatory within the program?		should it be mandatory?
			yes	no	
ID_1	editorial principles	no		x	no
ID_2	manual	yes	x		yes
ID_3	template	no		x	no
ID_4	template	yes		x	no
ID_5	manual	yes	x		yes
ID_6	manual	yes	x		yes
ID_7	manual	yes		x	uncertain
ID_8	template	yes		x	no
ID_9	manual	yes		x	uncertain
ID_10	manual	yes	x		yes
ID_11	manual	yes	x		yes
ID_12	template	no		x	yes
ID_13	editorial principles	yes		x	no
ID_14	methods report	yes	x		yes
ID_15	standard operating procedure	yes		x	yes
ID_16	methods report	yes	x		yes
ID_17	manual	no	x		yes

### Research question 2: influential factors on PVGs

#### Recurring challenges

**Fig. 3:**
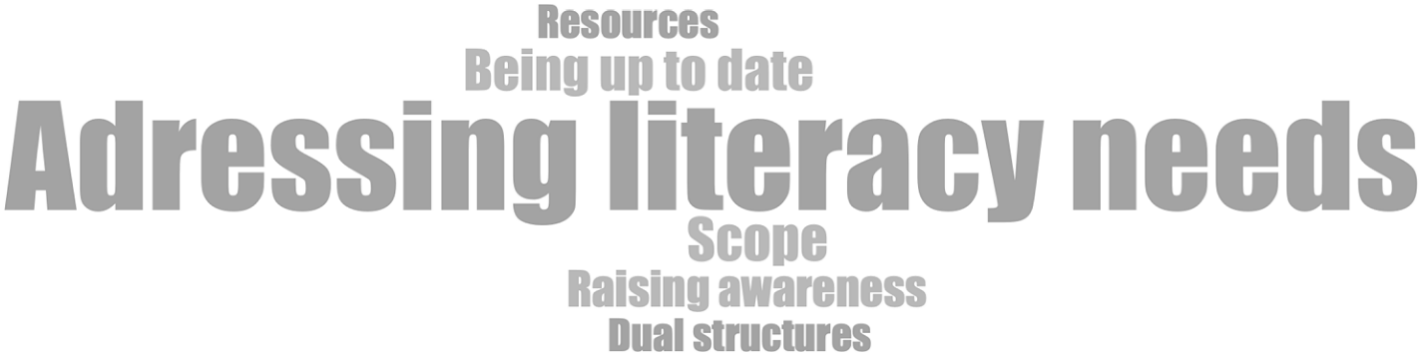
Recurring challenges in working with PVGs, size of words reflects frequency of occurrence

The challenges that respondents consistently face in working with PVGs are summarized in the terms listed in Fig. 3. Keeping information up to date, increasing awareness of PVGs, and the partial coexistence of different EBPI from different organizations can be challenging. The interviewees saw one major challenge in the handling of resources, e.g., personnel, time or financial resources. Meeting the literacy needs of diverse target groups is another challenge, not only in terms of lay language and comprehensibility of information, but also in taking into account individual and cultural needs.

I think appealing to broader cultural groups is important. So we’re not reaching perhaps some of the people who need it most. So refugees, immigrants, non-English [native] speaking language, people that will struggle with health information and understanding the health environment. (ID_4, Pos. 87).

#### Development, dissemination and implementation

Participants were asked about influencing factors, each related to the development, dissemination, and implementation of PVGs which are listed in Table 3. Although a well-structured approach may be a facilitator, an overly detailed methodology can also be seen as a disadvantage, as processes will be delayed and published information may no longer be up to date.

**Table 3:** Facilitating and hindering factors in developing, disseminating and implementing PVGs

	Facilitating factors	Hindering Factors
Development	clear methodological approach	too much methodology
	multidisciplinary team with clear responsibilities and experienced developers; including the patient perspective with trained representatives; strong networking “*(…) that’s why we also like to take the patients who have already participated. Because if they already know what a guideline is, what evidence-based medicine is, if they have already had a mini training course and already know the processes, then it is of course easier to work together with them.* (ID_14, Pos. 73)”	including the patient perspective and lack of methodological knowledge; working with volunteers; participation; disharmonies within the development group
	consultation and feedback	lack of consultation
	financial resources	lack of financial resources; prioritizing with limited resources; dependence on sponsors “*(…) So somebody, some organizations and groups, they have money, so they’ll give us money and say, “Would you do this one?” And so that’s quite a bit of a dilemma, I suppose, because it doesn’t mean that they’re more worthy. (…).* (ID_4, Pos. 55)
	good underlying CPG; mandatory development	compatibility of scope and evidence-base
		heterogeneous target group and addressing individuals at the same time; unclear aim of the product “*(…)But I think one of the key difficulties I think in translating recommendations, (…) they’re developed to a population level. And I think what people want from patient information is information that resonates with them as an individual.* (ID_1, Pos. 51).
Dissemination	various access routes; staff responsible for dissemination	lack of embedding in search engines; lack of awareness
	Co-branding, public relations and cooperation with stakeholder groups	lack of public relations; lack of professional media office
	Feedback mechanisms	lack of feedback
		lack of financial resources
		lack of actuality
Implementation	evaluation, audits	lack of research, discrepancy between recommendations and clinical reality
	various routes, staff responsible for implementation	lack of regulations on implementation
	“*(…)There are not many studies on this [implementation] yet, actually none at all. So I think there is still a lot to do. What is effective now, when you implement patient guidelines in everyday care.* (ID_7, Pos. 55)	

A multidisciplinary team was mentioned to be important, at best by representatives who have already been involved in developing the CPG or who are trained accordingly in advance. Although patient involvement was an essential aspect for all respondents, it can also present obstacles as it can be difficult when patient representatives lack sufficient methodological knowledge. Lack of resources, may it be time, personal or financial, were mentioned as big challenges. Furthermore, it can be difficult to address heterogeneous target groups and present information in an accurate and evidence-based manner while reducing the scope to improve readability.

As facilitators for dissemination, the interviewees mentioned using various access routes, through providing different formats of PVGs and also through public relations. Factors hindering dissemination were described as lack of public relations and professional media offices responsible for disseminating PVGs, lack of financial resources and a low awareness of PVGs in general.

For implementation, few answers were given as experiences on implementation of PVGs are lacking. One aspect seen as a barrier to implementation is the transfer of recommendations from the PVGs into everyday care.

### Future of PVGs

The participants emphasized that demographic change and digitalization are bringing about a change of information, so that in the future there will have to be a broader range of (interactive) formats and access options in order to reach all target groups, as “(.) *some patients (…) still want a printed copy”* but “the (…) focus should be on developing digital products and making them interactive. (ID_3, Pos. 73).

Due to an increasing number of elderly people, topics such as multi-morbidity, multi-medication and chronic diseases will gain focus in the future. However, an increased focus on culturally sensitive and easy-to-read inclusive information is also emphasized. The participants mentioned that from their point of view, PVGs or EBPI in general will only gain relevance.

(…) But I think those of us who are in the space of creating clinically relevant material, we have an obligation to make sure that we have the best information available that is accurate and informative and relevant. (…) I don’t think that the need for having this information is going to diminish, I think it’s only going to get greater (ID_15, Pos. 95)

**Fig. 4:**
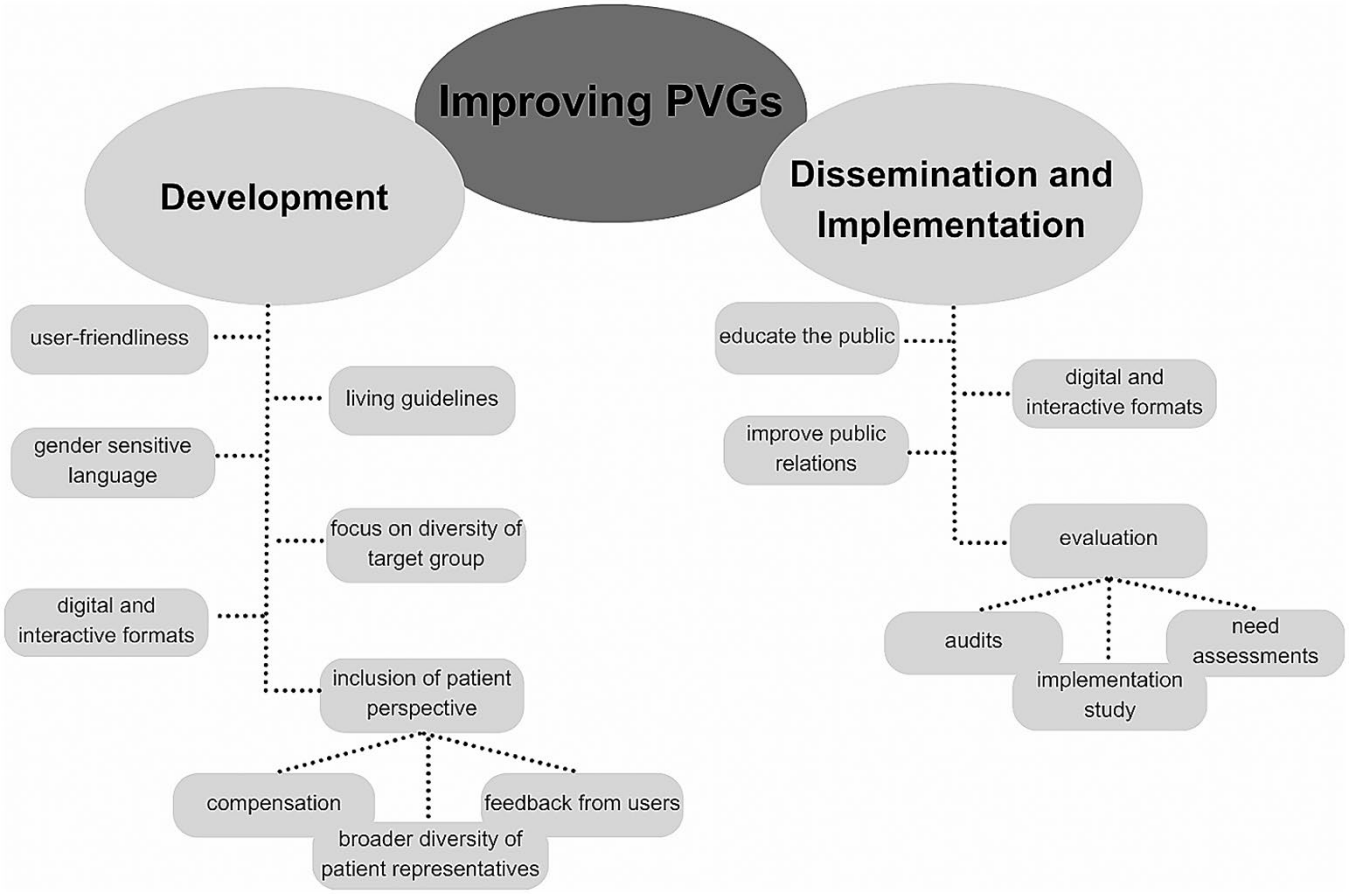
Ways to further improve the development, dissemination and implementation of PVGs

Figure 4 shows ways to further improve PVGs regarding their development, dissemination and implementation. One major planned and ongoing change in working with PVGs is keeping PVGs up to date.

(…) we’re trying to move very much towards what we call living guidelines. So, this constant cycle of update. (…) And I think that’s an issue for the future, is how do we make sure that patient guidelines are really responsive to the changes in evidence or the changes in recommendations? (ID_1, Pos. 73).

The interviewees said that there should be a stronger focus on including a diverse patient perspective and getting feedback, in order to improve the applicability of PVGs. As patient representatives usually work in the development group on a voluntary basis, compensation could be another way of improving patient involvement. They mentioned that providing different interactive formats is important to address diverse target groups and different levels of literacy. To improve dissemination and implementation of PVGs, raising awareness on their existence is key. Based on their experiences, participants suggested that evaluation on the usage of PVGs should be done through need assessments, audits or implementation studies.

## Discussion

The main issues that stood out during the interviews were raising awareness of PVGs in the first place, since many people who should be addressed by PVGs are often not even aware of their existence. To do so, using different kinds of formats, tools and dissemination channels in order to address target groups with different literacy needs were mentioned to be key. Patient involvement in the development and dissemination process is cited by the experts as probably the most decisive factor for the success of PVGs. The findings of the interviews are consistent with the results of other studies that patient involvement offers added value for research in general and also for CPGs [20, 21]. Nevertheless, the challenges of patient involvement, such as lack of resources and lack of training for lay members, are also congruent. Although patient and public involvement is considered to be of high importance, and most organizations involve patients at various levels the extent of involvement varies quite widely. While some already include patients and members of the public as part of the development group of a PVG, allowing for active participation in the PVG in the form of collaboration, pure consultation processes by patients or members of the public are considered more passive involvement [22]. Accordingly, the involvement of patients should not be considered a quality characteristic per se, and it should be critically questioned in principle when it is actually a stage of active participation or rather passive participation. As with dissemination, the use of different, parallel forms of lay member involvement in the development process of a PVG could be beneficial.

Although the relevance of patient involvement in the development of CPGs is now undisputed and demanded, the approaches and the extent of patient involvement in the CPG process still vary greatly in some cases and no standard approach for patient involvement in guideline development seems to exist [21, 23]. This is why the sometimes varying extent of patient involvement in the PVG process is not surprising. The 10-step framework for involving patients in the CPG development process, as proposed by Armstrong and colleagues, could potentially be applied in an adapted form for PVG development to introduce a standard approach to patient involvement [23].

German-speaking participants mentioned that within their programs, the development of PVGs is obligatory analogous to the CPG. They also follow the methodological guidelines of ÄZQ, OL and AWMF-IMWI [24] and publish a specific methods report for each PVG. PVGs are made available in Germany both as PDFs and as print versions, whereas on the international level, more “modern” formats are used such as interactive apps. International organizations seem to place less focus on detailed methodology, but rather on broad dissemination. As reported elsewhere [8, 11], the terminology used for PVGs varies, even in the English-speaking community, which is also highlighted by the 8 different terms identified in 17 interviews.

It can be concluded that although all participants consider PVGs as an important information tool, their development should be decided based on actual needs and resources rather than strictly mandating them. Since lack of resources was consistently mentioned as a hindering factor, encouraging the development of PVGs by providing adequate resources would be an important step toward improving information for patients.

Further, the interviews showed that experts in the development of PVGs have a wealth of experience, in some cases with different thematic priorities. Bringing this knowledge together, exchanging experiences in working with PVGs, learning from each other and agreeing on a minimum standard in the development of PVGs would be an important and, above all, transparent step in further developing PVGs. Having a collection of such consented standards could potentially even save resources, promote the use and recognition value of PVGs as well as the international exchange among experts.

Little information was available on the implementation of PVGs and how they are used in practice, as implementation studies usually are expensive. Information on implementation was almost impossible to obtain in this framework, as the experts interviewed were rather actively involved in the development and dissemination of PVGs. It is important to involve the target group of PVGs in particular and to strengthen feedback mechanisms in the future. Raising awareness of PVGs, one of the findings and key challenges identified in the interviews conducted, is also confirmed by findings from interviews conducted with healthcare providers and patients [25]. A further logical step would be to link the findings of the interviews with experts, patients and service providers and their views and experiences with PVGs in order to gain insights for the further development of PVGs.

### Limitations

A limiting factor is that the experts are involved in different areas of working with PVGs and have different levels of experience. If, for example, questions were asked about the methodology of PVGs, it must be assumed that not all of them can answer this question with certainty, and therefore a distortion of the statements arises.

As we only asked about aims and target groups but did not ask for a specific definition of PVGs within each organization, it may be difficult to compare the information on PVGs from different organizations. However, it became clear in all interviews that the PVGs were always based on CPG recommendations. In future studies, however, specific definitions of PVGs should be asked in advance in order to eliminate misunderstandings and enable comparability of the results.

Although we conducted interviews with participants from several different countries, due to the low response rate of international experts, we conducted more interviews on the German level than initially planned. As the German organizations who participated follow the same generic methods report [24] for developing PVGs, the congruent data of these interviews could cause a distortion of the findings.

## Conclusion

While the regulatory frameworks in the participating countries slightly differ, the steps in the PVG development process are largely similar. Focus is placed on the involvement of laypersons in the process, although the extent of participation varies. Even though there are increasingly more patient-directed knowledge tools, the experts collectively continue to attribute great importance to PVGs overall. In order to constantly adapt to medical progress and changing conditions, the focus in the future may be more on formats such as living guidelines. Digital or even interactive formats may be helpful in disseminating PVGs widely and reaching different target groups, however, the sufficient availability of resources was mentioned as an essential prerequisite. Although there are different views on the mandatory development of PVGs, there is a consistent call for more transparency regarding the methodology used for PVGs. This could be another step towards a future consensus on a methodological guide or at least minimum criteria for PVGs at the international level. Further, more research is needed on PVG implementation to understand how PVGs are used by patients and health care providers so that PVGs can be adapted accordingly in the future.

### Electronic supplementary material

Below is the link to the electronic supplementary material.

Supplementary Material 1

## Data Availability

The generated and analyzed datasets are not publicly available due to the need to protect participants’ privacy and confidentiality. These datasets are available from the corresponding author upon request.

## Abbreviations

AWMF: Association of the Scientific Medical Societies in Germany (German abbreviation)
ÄZQ: German Agency for Quality in Medicine (German abbreviation)
EBPI: evidence-based patient information
CPG: Clinical Practice Guidelines
GGPO: Office of the German Guideline Program in Oncology c/o German Cancer Society
PVG: Patient version of clinical guideline
