# Supplementary material for: Barriers and facilitators in developing patient versions of clinical practice guidelines - qualitative interviews on experiences of international guideline producers
Source: BMC Health Serv Res. 2024 Jan 16;24:78. doi: 10.1186/s12913-023-10524-5 (PMC10790536; doi:10.1186/s12913-023-10524-5)
Supplement: Supplementary file 1 — Supplementary Material 1 [file 12913_2023_10524_MOESM1_ESM.docx]

**Appendix A: COREQ (COnsolidated criteria for REporting Qualitative research) Checklist**

| **Topic** | | **Item No.** | **Guide Questions/Description** | **Reported on Page No.** |
| --- | --- | --- | --- | --- |
| **Domain 1: Research team and reflexivity** | | | | |
| *Personal characteristics* | | | | |
| Interviewer/facilitator | | 1 | Which author/s conducted the interview or focus group? | “*The interviewer (NM), who is a female health scientist, had little experience in conducting qualitative interviews but was trained in advance. The participants did not know the interviewer (NM), they only knew she was a researcher at Witten/Herdecke University*” |
| Credentials | | 2 | What were the researcher’s credentials? E.g. PhD, MD |  |
| Occupation | | 3 | What was their occupation at the time of the study? |  |
| Gender | | 4 | Was the researcher male or female? |  |
| Experience and training | | 5 | What experience or training did the researcher have? |  |
| *Relationship with participants* | | | | |
| Relationship established | | 6 | Was a relationship established prior to study commencement? | “*The participants did not know the interviewer (NM*), *they only knew she was a researcher at Witten/Herdecke University.*”  Participants received information about the AnImPaLLO study and its goal prior to the interview when invited to participate. No characteristics about the interviewer (NM) were reported, but she introduced herself at the beginning of every interview. |
| Participant knowledge of the interviewer | | 7 | What did the participants know about the researcher? e.g. personal goals, reasons for doing the research |  |
| Interviewer characteristics | | 8 | What characteristics were reported about the interviewer/facilitator? e.g. Bias, assumptions, reasons and interests in the research topic |  |
| **Domain 2: Study design** | | | | |
| *Theoretical framework* | | | | |
| Methodological orientation and Theory | | 9 | What methodological orientation was stated to underpin the study? e.g. grounded theory, discourse analysis, ethnography, phenomenology, content analysis | “*Sociodemographic data of the interviewees were descriptively processed using Excel 2016 and qualitative content analysis were performed using MAXQDA.”* |
| *Participant selection* | | | | |
| Sampling | | 10 | How were participants selected? e.g. purposive, convenience, consecutive, snowball | Section “*Study Population and Recruitment*” |
| Method of approach | | 11 | How were participants approached? e.g. face-to-face, telephone, mail, email |  |
| Sample size | | 12 | How many participants were in the study? |  |
| Non-participation | | 13 | How many people refused to participate or dropped out? Reasons? |  |
| *Setting* | | | | |
| Setting of data collection | | 14 | Where was the data collected? e.g. home, clinic, workplace | “*NM conducted the interviews either by telephone or ZOOM video conferencing, depending on time zones and the individual preferences of the interviewees.*” |
| Presence of non  participants | | 15 | Was anyone else present besides the participants and researchers? | no |
| Description of sample | | 16 | What are the important characteristics of the sample? e.g. demographic data, date | The sample is described in the *Results* section |
| *Data collection* | | | | |
| Interview guide | | 17 | Were questions, prompts, guides provided by the authors? Was it pilot tested? | *“The participants completed an informed consent form and a data protection statement and were provided with the interview guide in advance.”* |
| Repeat interviews | | 18 | Were repeat interviews carried out? If yes, how many? | *“No interviews were repeated and none had to be canceled”* |
| Audio/visual recording | | 19 | Did the research use audio or visual recording to collect the data? | “*The interviews were recorded using an audio recording device* (…).” |
| Field notes | | 20 | Were field notes made during and/or after the interview or focus group? | “*No field notes were made during the interview.”* |
| Duration | | 21 | What was the duration of the interviews or focus group? | “*The average duration of the interviews was 00:48:43 minutes (range 00:27:41 minutes to 01:19:56).”* |
| Data saturation | | 22 | Was data saturation discussed? | “*We planned to conduct interviews with the international organizations from a study on the content and purpose of PVGs by Santesso et al. and with national experts. We recruited until we reached data saturation.”* |
| Transcripts returned | | 23 | Were transcripts returned to participants for comment and/or correction? | *“In one case, transcripts were returned to participants because participants in a dual interview wanted to check for any misunderstandings that might have occurred due to the language barrier. Otherwise, no transcripts were sent for comment.”* |
| **Domain 3: analysis and findings** | | | | |
| *Data analysis* | | | | |
| Number of data coders | 24 | | How many data coders coded the data? | *“NM structured interview data deductively (…). JH then inductively refined the (…).”* |
| Description of the coding tree | 25 | | Did authors provide a description of the coding tree? | *“In addition, rules for coding and code specifications were defined (Appendix: data codes).”* |
| Derivation of themes | 26 | | Were themes identified in advance or derived from the data? | “*NM structured interview data deductively according to predefined main categories based on the core questions of the interview guide. JH then inductively refined the scheme with additional categories and subcategories.”* |
| Software | 27 | | What software, if applicable, was used to manage the data? | “*Sociodemographic data of the interviewees were descriptively processed using Excel 2016 and qualitative content analysis were performed using MAXQDA.”* |
| Participant checking | 28 | | Did participants provide feedback on the findings? | No |
| *Reporting* | | | | |
| Quotations presented | 29 | | Were participant quotations presented to illustrate the themes/findings? Was each quotation identified? e.g. participant number | yes, e.g. *“(...) even when you have people (...) who are health educators and have expertise in developing materials for the general public having someone who is a consumer to say, how they would interpret what you're trying to say is very important.” (ID_15, Pos. 99)* |
| Data and findings consistent | 30 | | Was there consistency between the data presented and the findings? | yes |
| Clarity of major themes | 31 | | Were major themes clearly presented in the findings? | yes, using subheadings |
| Clarity of minor themes | 32 | | Is there a description of diverse cases or discussion of minor themes? | yes; however, the findings were rather consistent |

Developed from: Tong A, Sainsbury P, Craig J. Consolidated criteria for reporting qualitative research (COREQ): a 32-item checklist for interviews and focus groups. *International Journal for Quality in Health Care*. 2007. Volume 19, Number 6: pp. 349 – 357

**Appendix B: Interview guide**

Good Morning/afternoon Ms/Mr(...)

First of all, I would like to introduce myself. My name is Nora Meyer and I work at the Institute for Research in Operative Medicine (IFOM) at the University of Witten/Herdecke in Cologne. I am very happy that you support our research project by participating in this interview. Do you have any questions or comments before we start?

Your individual experience and expertise in the context of patient guidelines are of great interest to us. Accordingly, I would like to point out that there are no wrong answers in the interview. Please let me know if there is a question you do not want to answer. If there is something you do not understand in terms of content or acoustics, please also let me know and I will repeat this section.

As already mentioned in advance, the interview will be recorded with a recording device. You have already given your written consent to this. I will now turn on the device [turn on recording device] and ask you first, to have this on tape, "Do you agree to the recording of the interview?” [yes/no]

• **General Information***For the purpose of comparability with other interview partners, I will first ask you some general*

*questions about yourself.*

- I note that you are female/male, is that correct? [Note gender]
- Would you tell me your age? [Note age]
- Would you please tell me your highest level of education?
- Would you tell me the position in which you are currently employed?
- How long have you been working in this position?
- How many patient guidelines have you been involved in writing?
- How many years of experience in creating patient guidelines do you have?
- What are the main topics of the patient guidelines you have been involved in developing so far?

• **Guideline-related information**

*Please answer the following questions about your work with patient guidelines.*

- There are different terminologies around the world. What does your organization call patient guidelines? (e.g. patient versions, patient information, public version...)
- What is your organization's primary aim in developing patient guidelines?
- In which formats are your patient guidelines available? (Print, PDF, App)
- Are you working on specific projects for further improvement of your patient guidelines or are any in planning? (process efficiency, dissemination, implementation, digitization, ...)

o IF YES: which ones?

- Are there any recurring challenges in working with patient guidelines? o IF YES: Would you please explain this more detailed?

• **Role of patient guidelines**

*In the following two sections, I would like to ask you questions about the role and target group of patient guidelines.*

- For which target groups do you primarily design the patient guidelines?
- In your view, what is the significance of patient guidelines for patients, relatives and health care providers?
- How is the patient perspective ensured in the development of patient guidelines?
  o If not mentioned: What role do you ascribe to the involvement of patient representatives in the process of developing patient guidelines?
- In your opinion, is there a need for improvement in your patient guidelines with regard to different target groups?

o If yes, at which point? (Scope, format, design and presentation, comprehensibility of content, (recommendations), relevance and completeness of information)

• **Factors influencing patient guidelines***In this section, I would like to discuss factors that influence the process from development to*

*implementation of patient guidelines, both positively and negatively.*

**Development**

• In your experience, what factors promote the development of patient guidelines in the first place?

•  In your experience, which factors have a positive effect on the process of developing patient guidelines?

In your experience, which factors have an unfavorable effect on the process of developing patient guidelines?

**Dissemination**

- In your experience, what factors are beneficial for the dissemination of patient guidelines?
- In your experience, what factors hinder the dissemination of patient guidelines?

**Implementation**

- In your experience, which factors are beneficial for the implementation of patient guidelines?
- In your experience, what factors hinder the implementation of patient guidelines?
- In your opinion, what can be improved in the process from the development to the implementation of patient guidelines?

o What could such improvements look like from your point of view?

• **Methodology of patient guidelines**

*In this section, I would like to talk about the methodological approach to developing patient guidelines.*

- Can you briefly describe the main methodological steps of the procedure for the development of patient guidelines in your organization?
- To what extent does your organization have internal directives or uniform standards for the methodological approach to the development of patient guidelines?
  Examples would be a (generic) methods report, checklist, etc.

o If available, please briefly explain these internal directives.

If not yet mentioned (if necessary, differentiate between generic and specific method report):

- Is a description of the methodology accessible for the public?
- Is this description of the methodology also presented in a way that is understandable for patients?

o IF NOT: To what extent do you consider this useful?

In Germany, in the National Health Care Guidelines (NVL) program and in the Oncology Guidelines

Program (OL), patient guidelines are a mandatory component of medical guidelines.

- To what extent is the development of patient guidelines mandatory in your country?
  o IF NOT: To what extent should the development of patient guidelines be mandatory in your country?
  o What advantages and disadvantages could this have from your point of view?

• **Outlook**

*This is the final section. Here, I would like to venture into an outlook into the future. Our society is in a constant state of change, also regarding health and disease and the factors that influence them. Two phenomena are frequently brought up in discussions of current and, above all, future social challenges and potentials: demographic change and digitalization.*

- With reference to patient guidelines, how do you assess the possible influence of demographic change in the future?
- With reference to patient guidelines, how do you assess the role of digitalization in the future?
- In your opinion, to what extent will the relevance of patient guidelines change in the future?
- In your view, are there still important aspects in the process of developing to implementing patient guidelines that have not yet been addressed?

**Conclusion**

I've now come to the end of the interview. Is there anything else you would like to mention or add to the interview?

I will now turn off the recorder. [Turn off recording device]

Following the project, would you like to be informed of the results?
IF YES: How would you like to be contacted? [record contact information]

On behalf of everyone involved in the project, thank you very much for your participation, your expertise, and your time!

**Appendix C: Data codes**

| **Content** | **Category** | **Subcode** | **Rule** | **Specification** |
| --- | --- | --- | --- | --- |
| General Information  (black) | Gender | Female | one word | Female/Male or not specified, if participants did not answer the question |
|  |  | Male |  |  |
|  |  | Not specified |  |  |
|  | Age |  | numbers | Years |
|  | Education | ISCED >5 | one word | University degree or technician training = high level of education (ISCED > 5); vocational training or higher education entrance qualification = medium level of education (ISCED 3/4). neither completed vocational training nor higher education entrance qualification = low level of education (ISCED < 3)^[[1]](#footnote-1)^ |
|  |  | ISCED 3/4  (not applicable) |  |  |
|  |  | ISCED <3  (not applicable) |  |  |
|  | Current position |  | one term | Actual current occupation, not learned profession |
|  | Experience in position |  | numbers and unit | Time in years |
|  | Number of PVGs |  | numbers | Number of PVGs with personal involvement. Can be any involvement in working with PVGs^[[2]](#footnote-2)^ |
|  | Experience with PVGs |  | numbers and unit | Time in years, can be any experience (independent of current position) |
|  | Terminology |  | one term | Country- or organization-specific terminology used for PVGs (only applies to international interviews) |
| General questions about PVGs  (yellow) | Aims |  | context | Aim/goal of PVGs, either of organization or individual |
|  | Contents | Thematic contents | context | Can be both: topics/thematic contents vs. contents in terms of structure (International interviews: topics of PVGs involved, National interviews: actual contents usually found in patient versions) |
|  |  | Structural contents |  |  |
|  | Formats |  | context | Available formats published by organization (PDF, print, …) |
| Questions about target group  (pink) | Target group |  | context | Listing of target group(s) the PVGs are designed/aimed for |
|  | Significance of PVGs for target group |  | context | Importance of PVGs for target group from the expert's point of view (speculative question) |
|  | Patient perspective ensured |  | context | How patient perspective is ensured in the development of PVGs |
|  | Role of patient representatives |  | context | Role of the involvement of patient representatives in the process of developing patient versions (speculative question) |
| Methodology of PVGs  (purple) | Initiation of a PVG |  | context | Launch of developing a PVG (kick-off), how the process starts, who decides on the development of a PVG |
|  | Methodological approach | Prioritization of recommendations | context | Description of the main methodological steps in the development of a PVG within the organization, decision on which recommendations to include in the PVG, how recommendations from CPG are selected to be included into the PVGs, structure or process of prioritization |
|  | Internal standards/methods report | Accessibility / Extent | context | Public accessibility of internal standards/methods report on the development of PVGs and the extent to which there are internal specifications or directives for the methodological procedure in the development of PVGs within the organization |
|  |  | Layman's terms | context | Presentation of a publicly available description of the methodology in a form that can be understood by laypersons |
|  | Mandatory development of PVGs |  | context | Is the development of PVG mandatory as in the German Guideline Program Oncology? Possible advantages and disadvantages of obligation |
| Future of PVGs  (blue) | Change of relevance |  | context | To what extent will the relevance of PVGs change in the future (speculative question) |
|  | Demographic change |  | context | Expected impact of demographic change on PVGs (speculative question) |
|  | Digitalization |  | context | Expected impact of digitalization on PVGs (speculative question) |
|  | Missing aspects |  | context | Any aspects that are not yet addressed in the whole process from development to implementation of PVGs or that are not sufficiently addressed. |
| Challenging/Influential factors on PVGs  (green) | Development | Barriers | context | Experts' perceptions about barriers, facilitators or in general influencing factors in the development of PVGs |
|  |  | Facilitators |  |  |
|  | Dissemination | Barriers | context | Experts' perceptions about barriers, facilitators or in general influencing factors in the dissemination of PVGs (may be speculative as experts may not have experience or insight into the dissemination) |
|  |  | Facilitators |  |  |
|  | Implementation | Barriers | context | Experts' perceptions about barriers, facilitators or in general influencing factors in the implementation of PVGs (may be speculative as experts may not have experience or insight into the implementation) |
|  |  | Facilitators |  |  |
|  | Challenges | Literacy needs / Language | context | Any recurring challenges in working with PVGs, either addressing patient’s literacy needs / language (e.g., challenge to write in plain language), scope of PVGs (e.g. versions are too long), funding (e.g., voluntary authors) or any other challenge that cannot be assigned to any of the categories |
|  |  | Scope |  |  |
|  |  | Funding |  |  |
|  |  | Other |  |  |
| Changes  (brown) | Projects for advancing PVGs |  | context | Any institutional/organizational projects for advancing PVGs (ongoing or planned) (e.g., process efficiency, dissemination, implementation, digitization, ...) |
|  | Suggested improvements |  | context | Experts' perceptions on specific need for improving PVGs (CAVE: Distinction from challenging factors: Here it is a matter of clearly defined suggestions for improvement, wishes, expectations) |

1. UNESCO Institute for Statistics. International Standard Classification of Education ISCED 2011. ISBN 978-92-9189-123-8, URL: https://uis.unesco.org/sites/default/files/documents/international-standard-classification-of-education-isced-2011-en.pdf; 2012. [↑](#footnote-ref-1)
2. patient versions of clinical practice guidelines [↑](#footnote-ref-2)
